# Supplementary figures and images for: Uterine Commensal Peptostreptococcus Species Contribute to IDO1 Induction in Endometrial Cancer via Indoleacrylic Acid
Source: Biomedicines. 2024 Mar 4;12(3):573. doi: 10.3390/biomedicines12030573 (PMC10968020; doi:10.3390/biomedicines12030573)

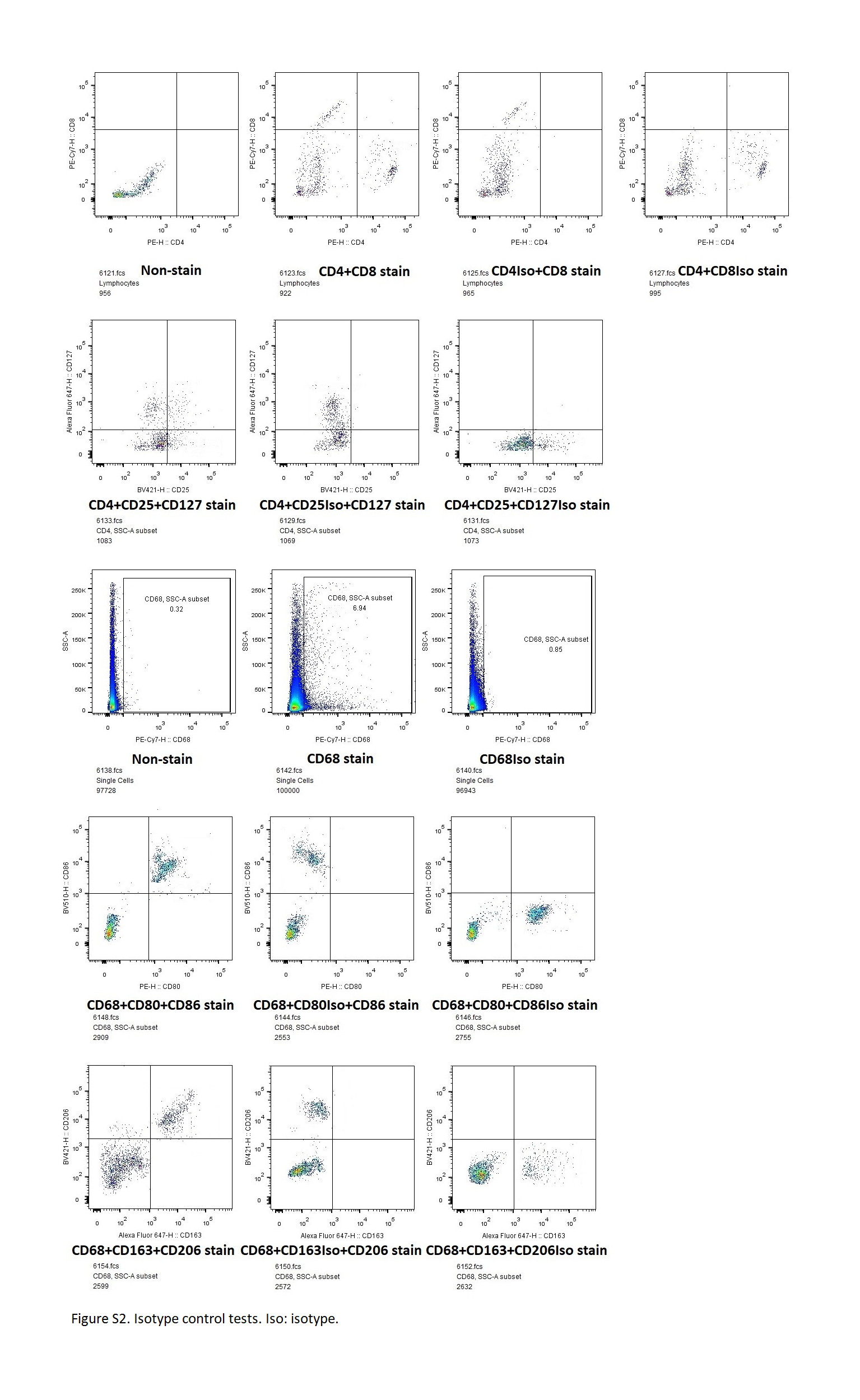

Supplement: Supplementary file 1 [file biomedicines-12-00573-s001.zip › figure S2 300dpi.tif]

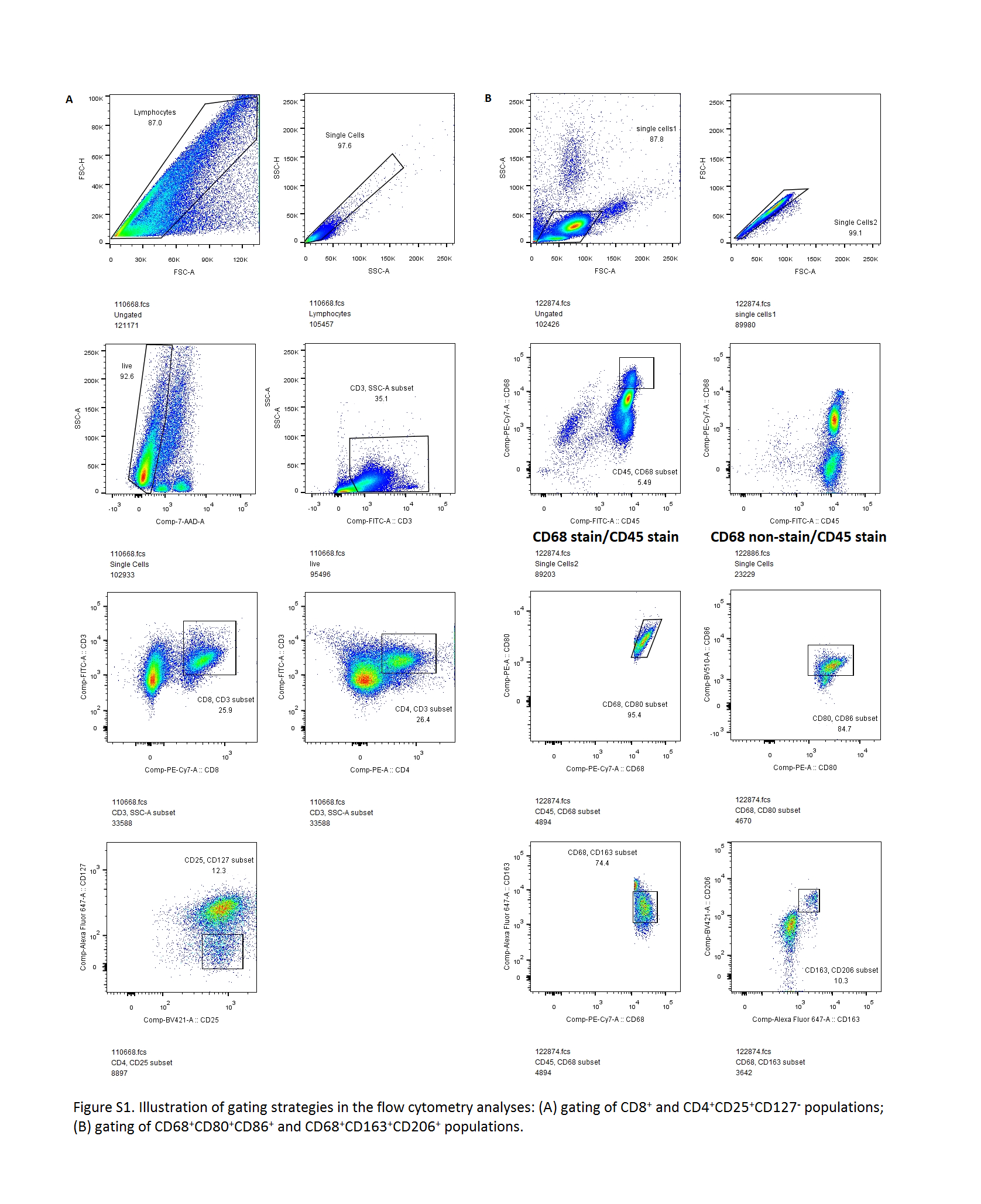

Supplement: Supplementary file 1 [file biomedicines-12-00573-s001.zip › figure S1 300dpi.tif]
